# Electroencephalographic Workload Indicators During Teleoperation of an Unmanned Aerial Vehicle Shepherding a Swarm of Unmanned Ground Vehicles in Contested Environments

**DOI:** 10.3389/fnins.2020.00040

**Published:** 2020-02-14

**Authors:** Raul Fernandez Rojas, Essam Debie, Justin Fidock, Michael Barlow, Kathryn Kasmarik, Sreenatha Anavatti, Matthew Garratt, Hussein Abbass

**Affiliations:** ^1^School of Engineering & IT, University of New South Wales, Canberra, NSW, Australia; ^2^Defence Science and Technology Organisation, Adelaide, SA, Australia

**Keywords:** augmented intelligence, cognitive load, human-autonomy teaming, human-swarm teaming, shepherding, mental load, cognitive indicators, EEG

## Abstract

**Background:** Although many electroencephalographic (EEG) indicators have been proposed in the literature, it is unclear which of the power bands and various indices are best as indicators of mental workload. Spectral powers (Theta, Alpha, and Beta) and ratios (Beta/(Alpha + Theta), Theta/Alpha, Theta/Beta) were identified in the literature as prominent indicators of cognitive workload.

**Objective:** The aim of the present study is to identify a set of EEG indicators that can be used for the objective assessment of cognitive workload in a multitasking setting and as a foundational step toward a human-autonomy augmented cognition system.

**Methods:** The participants' perceived workload was modulated during a teleoperation task involving an unmanned aerial vehicle (UAV) shepherding a swarm of unmanned ground vehicles (UGVs). Three sources of data were recorded from sixteen participants (*n* = 16): heart rate (HR), EEG, and subjective indicators of the perceived workload using the Air Traffic Workload Input Technique (ATWIT).

**Results:** The HR data predicted the scores from ATWIT. Nineteen common EEG features offered a discriminatory power of the four workload setups with high classification accuracy (82.23%), exhibiting a higher sensitivity than ATWIT and HR.

**Conclusion:** The identified set of features represents EEG indicators for the objective assessment of cognitive workload across subjects. These common indicators could be used for augmented intelligence in human-autonomy teaming scenarios, and form the basis for our work on designing a closed-loop augmented cognition system for human-swarm teaming.

## 1. Introduction

Mental workload refers to the depletion of mental resources due to mental demands imposed by a task on an individual. When task difficulty increases, mental workload increases due to the reduction in available cognitive resources. Research has shown that when an individual is under high cognitive workload and the cognitive workload approaches the individual's cognitive capacity, suboptimal decisions and human errors are expected. In the absence of any increase of task demand, prolonged mental activities also leads to depletion of cognitive resources (Kamzanova et al., 2014). Low workload can also lead to errors, due to boredom and the possibility for human distraction from the main task due to environmental influencing factors.

Humans have a limited amount of resources (both physically and mentally); therefore, optimizing these resources toward specific sets of tasks is likely to produce better results. However, it is challenging to understand these human limitations within a work environment due to many factors, such as demographic factors (gender, age, ethnicity), intrinsic motivation, mood states (happy, sad, anxious, etc.), previous experience, and different problem-solving strategies due to mental abilities, education, and skills. For example, the level of difficulty to accomplish a task might be seen differently by two operators; operator A could see the task difficult at first, but then find a good strategy to solve the task, while operator B could find the task extremely difficult, get discouraged, and fail to complete the task. As human resources are limited, there is a problem when a task demands more resources (Maior et al., 2014).

In many domains, the ability to process information, to react to different environments, and to make accurate decisions is vital. For instance, air traffic controllers (ATCs) generally perform in a highly cognitively-demanding environment, working for long periods of time, and under stress (Dasari et al., 2017). This scenario can lead to depletion of cognitive resources and thus degradation of performance. Another clear example is doctors and nurses in critical care units, they face large volumes of work, need to act quickly, and stay alert after many hours of intense work. In this case, errors and compromised standards signify that quality and safety of patient care might be endangered (MacPhee et al., 2017). It is, therefore, evident that there is a need to measure mental workload to identify the changes of cognitive demands on an individual while completing a task, which can potentially help reduce errors, task failure, accidents, and thus improve and maintain performance longer.

A number of metrics have been proposed for measuring mental workload. In the literature, these metrics can be divided into two main groups: subjective and objective measures. Subjective metrics are based on an operator's opinions, answers to questionnaires, and interviews. A popular technique for the subjective assessment of an operator's mental workload is the NASA Task Load indeX (NASA-TLX) (Hart and Staveland, 1988). This method uses six dimensions: mental demand, physical demand, temporal demand, performance, frustration level, and effort, each with 10- or 20-point scale. An overall rating is then calculated as the weighted mean of all six ratings. One of the limitations of NASA-TLX is the lack of continuous measurement while the task is performed, since participants typically answer the survey questions after a task is completed and they may be unable to recall the workload experienced during a trial. The Air Traffic Workload Input Technique (ATWIT) (Stein, 1985) is less pruned to this problem. Although, it is a workload rating scale designed for use in air traffic control studies, it has been successfully applied in other domains (Loft et al., 2015). This technique uses a scale from 1 (low workload) to 7 (high workload), which is administered by freezing the simulation. At each freeze, participants are asked to report their level of workload. An advantage of using this technique is that it enables a more accurate evaluation, since the participant can report the workload as it changes, instead of waiting until the end of the task/scenario to report workload.

Objective measures are generally based on experimental methods used to collect physiological and/or behavioral information by a single sensor or a combination of different types of sensors, simultaneously (Debie et al., 2019). In contrast with subjective measures, objective techniques offer a continuous measure of workload in real time, and also their implementations do not interfere with the performance of the task at hand (Wang et al., 2015). In general, objective measures can be classified either as neurophysiological, physiological, or behavioral. Neurophysiological measures include electroencephalography (EEG) and functional near-infrared spectroscopy (fNIRS) (Hirshfield et al., 2009). Physiological measures include electrocardiography (ECG) (Veltman and Gaillard, 1996), heart rate and heart rate variability (HRV) (Elkin-Frankston et al., 2017), pupil dilation (Pomplun and Sunkara, 2003), blink frequency and blink duration (Tsai et al., 2007), and saccades (Ahlstrom and Friedman-Berg, 2006). Behavioral measures include keystroke dynamics, mouse tracking, and body positioning (Mota and Picard, 2003). Most objective measures (physiological and neurophysiological) rely on the assumption that changes in cognitive demands are reflected in the autonomic nervous system (ANS) (Mulder, 1989; Veltman and Gaillard, 1996). Although, physiological measures can be used as indicators of mental workload, neurophysiological techniques are considered the most direct indicators of different cognitive states (Debie et al., 2019).

There are two main techniques with appropriate temporal resolution to measure cognitive workload using brain signals: fNIRS and EEG. fNIRS measures cognitive workload by examining the levels of oxygenated (HbO) and deoxygenated (HbR) hemoglobin concentration in the cerebral cortex (Rojas et al., 2017b), and alertness, and indicative of loss of cortical arousal (Kamzanova et al., 2014). In this regard, fNIRS is commonly used to measure the amount of effort exerted in a given brain region in response to a given task. Different studies have reported that increased levels of HbO in the pre-frontal cortex correlates with increased task engagement which is used to indicate increased cognitive workload (Ayaz et al., 2012; Herff et al., 2014). On the other hand, EEG measures the brain's electrical activity and pattern analysis of this activity is used to indicate different levels of cognitive workload. Spectral analysis is used to decompose EEG signals into their constituent frequency components. Typically, EEG data are partitioned into five bands (from slowest to fastest: delta, theta, alpha, beta, and gamma). The power spectral density (PSD) in each band is computed and used to compare the conditions being studied (i.e., low vs. high workload). EEG is considered the most popular approach in the literature to objectively assess cognitive states (Gevins et al., 1997; Abbass et al., 2014; Dong et al., 2016; Rojas et al., 2019a).

Although many indicators have been proposed in the literature, it is unclear which of the power bands and various indices is the most optimal for mental workload. In the following section, we present the most prominent indicators in the literature and their relationship with cognitive workload. The intent is not to provide an exhaustive literature review, but identify EEG metrics that could be potentially used as indicators of mental workload in our experiment.

### 1.1. EEG Indicators of Mental Workload

In the literature, EEG correlates of spectral powers at different cortical locations have been proposed for the assessment of cognitive workload. For example, theta band (4–8 Hz) has been linked to mental fatigue and mental workload (Gevins et al., 1995). Theta spectral power is thought to increase with increase demands on cognitive resources (Vidulich and Tsang, 2012; Xie et al., 2016), with higher task difficulty (Antonenko et al., 2010), and with increase of working memory (Borghini et al., 2012); particularly, theta power increases in tasks requiring a sustained concentration (Gevins and Smith, 2003). In addition, increase in theta power is related to lower mental vigilance and alertness, and indicative of loss of cortical arousal (Kamzanova et al., 2014). An increase in theta power monitored over the frontal cortex has been linked to an increase in task difficulty and use of higher memory resources (Parasuraman and Caggiano, 2002), frontal theta also increases during vigilance (Paus et al., 1997).

Alpha band (8–12 Hz) power has shown sensitivity to experiments in mental workload (Sterman and Mann, 1995; Xie et al., 2016; Puma et al., 2018), cognitive fatigue (Borghini et al., 2012), and also with reduction in attention or alertness (Kamzanova et al., 2014). In general, alpha band increases in relaxed states with eyes closed and decreases when the eyes are open (Antonenko et al., 2010). An increase in alpha power is related to lower mental vigilance and alertness (MacLean et al., 2012; Kamzanova et al., 2014) and therefore a decrease in the attention resources allocated to the task (Vidulich and Tsang, 2012). On the other hand, a progressive suppression of alpha waves has been linked to increasing levels of task difficulty (Mazher et al., 2017). Cortical areas that have been associated with alpha band changes are parietal and occipital areas (Dasari et al., 2017; Puma et al., 2018).

Beta band (12–30 Hz) has been linked to visual attention (Wróbel, 2000), short-term memory (Tallon-Baudry et al., 1999; Palva et al., 2011), and hypothesized to react to an increase in working memory (Spitzer and Haegens, 2017). An increase in beta power is associated with elevated mental workload levels during mental tasks (Coelli et al., 2015) and concentration (Kakkos et al., 2019). In addition, beta band activity reflects an arousal of the visual system during increased visual attention (Wróbel, 2000). An increase in beta activity has been observed in the parieto-occipital channels during visual working memory tasks (Mapelli and Özkurt, 2019).

In addition, the use of multiple EEG frequency bands (ratios or indices) has been proposed as an indicator of mental workload. This is based on the assumption that by combining information from multiple bands, the assessment of workload can be enhanced. For example, beta/(alpha + theta) (or Engagement Index, EI) has been used to study alertness and task engagement (Pope et al., 1995; Freeman et al., 1999; Mikulka et al., 2002), mental attention investment (MacLean et al., 2012), and mental effort (Smit et al., 2005). When alpha reduction was observed to correlate with increases in activity in frontal-parietal cortical areas, beta power increased while theta decreased, indicating a state of high vigilance (MacLean et al., 2012). When alpha reduction was seen to correlate with increases in activity in occipital and parietal areas, beta decreased and theta increased, indicating a state of drowsiness, or low vigilance (MacLean et al., 2012).

Another index used to explore the assessment of workload is the theta/alpha ratio (or Task Load Index, TLI). This index is based on the assumption that an increase of mental load is associated with a decrease in alpha power and an increase in theta power (Stipacek et al., 2003; Käthner et al., 2014). While an increased level of fatigue is related to increase of alpha and theta powers (Käthner et al., 2014; Xie et al., 2016). Research has shown that workload manipulations increased theta power at anterior frontal and frontal midline regions and decreased alpha power at parietal regions (Gevins and Smith, 2003). In general, an increase of cognitive workload has been associated with an increase of theta power together with a decrease of alpha power (Fairclough and Venables, 2004).

Theta/beta ratio has been used to study attention-deficit/hyperactitivty disorder (ADHD) and working memory problems in children (Lansbergen et al., 2011). This ratio shows increased theta power and decreased beta power during resting state in individuals with ADHD (Barry et al., 2003). Theta/beta ratio has been negatively correlated with mean reaction time in adults, indicating an increased theta/beta ratio linked to shorter, faster reaction time (Loo and Makeig, 2012). Theta/beta ratio has been used for monitoring sleepiness and wakefulness in car drivers (Sun et al., 2015). This ratio has been used to discriminate distraction from attentive driving as measured in the parietal lobe (Zhao et al., 2013). This index is based on the assumption that an increase in alertness and task engagement results in an increase in beta power and a decrease in theta power (Gale and Edwards, 1983). Table 1 presents a summary of EEG indicators for the assessment of cognitive workload identified in the literature.

**Table 1:** Summary of EEG correlates of spectral powers for the assessment of cognitive workload in the literature.

**Indicator**	**Type of cognitive**	**Description**
	**behavior**	
*Theta*	Workload, vigilance, and concentration.	Theta spectral power is thought to increase with increase cognitive resources demand.
		Theta increases in tasks requiring a sustained focus of concentration and vigilance.
*Alpha*	Workload, cognitive fatigue, and attention.	Alpha band increases in relaxed states with eyes closed and decreases when the eyes are open.
		An increase in alpha power is related to lower mental vigilance and alertness.
*Beta*	Workload, visual attention, and concentration.	An increase in beta power is associated with elevated mental workload levels during mental tasks and concentration.
		Beta band activity reflects an arousal of the visual system during increased visual attention.
$\frac{Beta}{Alpha+Theta}$	Mental Effort, vigilance, and attention.	It has been used to study alertness and task engagement, mental attentional investment, and mental effort.
$\frac{Theta}{Alpha}$	Workload, mental effort.	This index is based in the assumption that an increase of mental load is associated with a decrease in alpha power and an increase in theta power.
$\frac{Theta}{Beta}$	Working memory, attention, and sleepiness.	This index is based in the assumption that an increases in alertness and task engagement result in an increase in beta power and a decrease in theta power.

The present study was conducted to directly address the challenge to identify a set of indicators that can be used for the objective assessment of cognitive workload in a multitasking setting. Consequently, we have designed a simulation environment which affords manipulation of task complexity by varying the quality of information in the simulation. It has been shown that information quality affects cognitive workload (Young et al., 2016). Finally, we aim to identify EEG indices that may be used to trigger technological support to maintain performance.

## 2. Methods

### 2.1. Participants

Sixteen participants (four females) were recruited. Their age ranged from 22 to 50 years old (mean age 33 ± 8.1 std). The experiment was approved by the University of New South Wales (UNSW) Research Ethics Committee (protocol ID: HC180554). All participants provided written informed consent prior to participating in the study. A demographics questionnaire was given to the participants before the start of the experiments. Participants did not receive monetary compensation for their participation in this study.

### 2.2. Description of the Experiment

Participants were seated on a fixed chair in front of a computer screen placed on a desk. An introduction to the experimental procedure and a practice session were provided to the participants before the start of the study. After that, the EEG head cap was mounted on the participants' head. To minimize any muscle movement artifacts, the participants were instructed to remain as still as possible while holding the mouse at all times during the experiment. Next, a 1 min baseline recording was obtained, in the first 30 s, the participants were told to close their eyes; then in the remaining 30 s, the participants were told to keep their eyes open and fixed on a point in the center of the screen. Finally, the participants were instructed to start the experiment after a 2-min break; the complete session lasted ~50 min.

**Figure 1:**
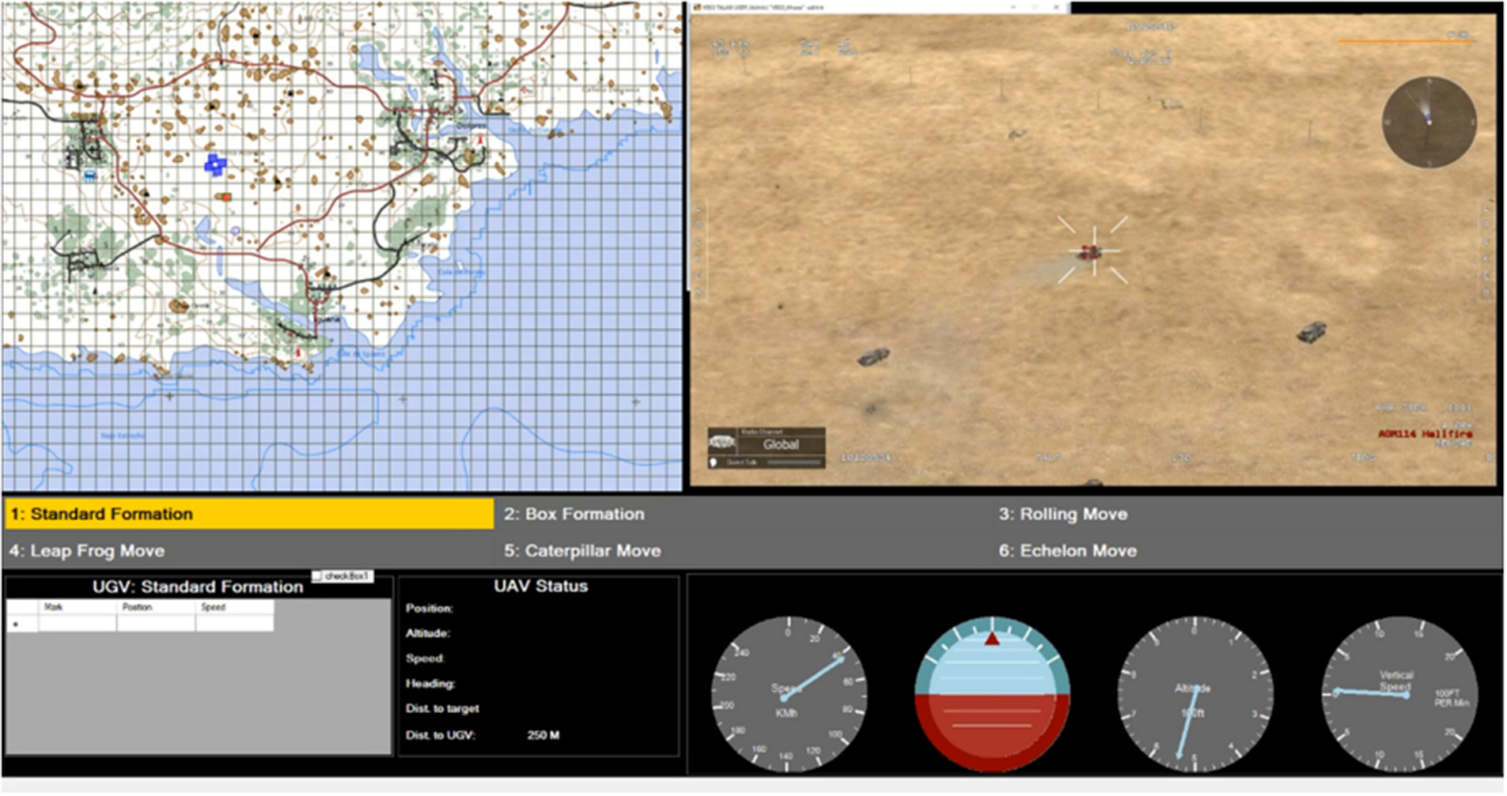
UAV pilot interface.

The experimental task was to teleoperate an unmanned aerial vehicle (UAV) to guide a swarm formation of autonomous unmanned ground vehicles (UGVs). Only the UAV remote-operator knows the destination defined by the mission profile. The UGVs consist of a group of four vehicles with capabilities to self-organize to autonomously maintain a formation during the mission. The operator's graphical user interface (presented in Figure 1) displays sufficient information to successfully guide the UAV with information display on the UAV (e.g., speed, altitude), mission state information, navigation map, and localization of the UGVs. This experiment is designed to run the simulation that combines four scenarios of different levels of information quality. Each scenario lasts 4 min and is repeated two times per participant. Simultaneously, EEG data and heart beat were recorded continuously during the experimental task. During each experimental condition, participants rated their mental effort using a computer version of the ATWIT questionnaire.

### 2.3. Simulation Environment

The experimental task is undertaken using the Virtual Battlespace Simulation 3 (VBS3) (Bohemia Interactive Simulations, Orlando Florida, USA) environment. The VBS3 software was used under the Australian Defence Force (ADF) Enterprise Licence Agreement with BISSimulation Australia. Information latency and loss were modeled to impact the operator's control station (as illustrated in Figure 1). This interface was programmed in C# (Microsoft Corporation, Washington, USA) since VBS3 does not have the capability for simulating information latency and loss as required. The interface has two main graphic displays located side by side on the top. On the left side, there is a lateral view of the UAV and UGVs' positions on a map. The UAV is presented by a green rectangle and the UGVs are visualized as blue rectangles. A blue star marks the UGVs' destination on the map. On the right side, real-time video streamed from the UAV camera is provided to the operator. At the bottom of the interface, detailed textual information on the UAV and UGVs' status including their positions, headings and speeds are provided. In the middle of the interface, a panel lists all possible UGV formation options; however, for this study we limit the formation to a boxing formation alone.

### 2.4. Experimental Design

A within-subject design with four different experimental conditions determined by the levels of quality of information was used in this study. The four experimental conditions (scenarios) are: (1) low latency/delay and low dropout; (2) low delay and high dropout; (3) high delay and low dropout; and (4) high delay and high dropout. The experiment is counterbalanced by using the composite 3 × 3 Latin Square design to avoid confounding due to order effects. In our experiment, information latency is the amount of time a video frame from the UAV camera and the status of all vehicles to traverse in the camera' s field of view are delayed to the interface; while, information loss is the rate in which video frames and data about the status of vehicles are not transmitted during data transmission. Ideally, information latency should be unnoticeable to the UAV operator and the delivery of information should be operationally assured.

However, to modulate the participants' perceived workload, information latency and information loss are injected into the simulation. Thus, it has been hypothesized that the latency and loss of information affect the subjects' perceived cognitive workload. Information latency and information loss are modeled using two parameters, *d* for the delay time (Low *d* = 1 s, High *d* = 9 s) of information transmission, and *lf* for the number of video frames lost per second (Low *lf* = 1 s, High *lf* = 9 s) in transmission. Table 2 lists the parameter values corresponding to the corresponding levels of information latency and loss, respectively.

**Table 2:** Variables used in information latency and loss.

**Variable**	**Level**	**Parameter value**
Information latency	Low	*d* = 1 s
	High	*d* = 9 s
Information loss	Low	*l*_*f*_ = 1 s
	High	*l*_*f*_ = 9 s

### 2.5. Heart Rate Measurement

A mouse (Mionix Naos QG) equipped with heart rate (HR) and galvanic skin response (GSR) sensors is used as the main input method during the simulation. The biometric sensors are designed to measure the physiological data on the palm of the user; thus, the user must maintain the mouse in their hand at all times while the simulation is running. The mouse uses a sample rate of eight samples per second. In addition, the mouse can also record different mouse metrics, such as: number of scrolls, clicks, and movements. In this study, the heart rate information is only used to corroborate the design of the experimental conditions due to its high sensitivity to mental load measure (Cinaz et al., 2010).

### 2.6. Electroencephalographic (EEG) Measurement

**Figure 2:**
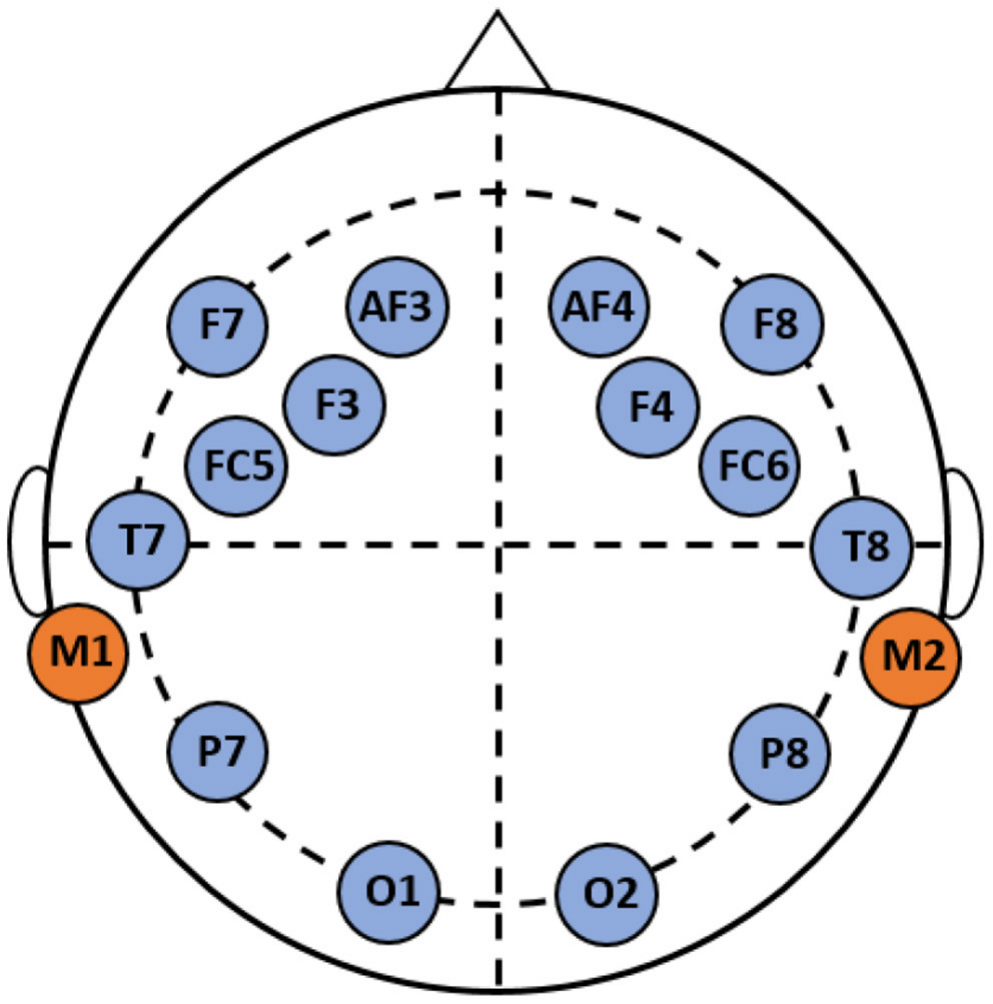
Cortical areas covered by the electrodes of the EEG EMOTIV Epoc.

A wireless EEG acquisition system (Emotiv EPOC) was used to record neural activity. This device has a resolution of 14 channels (plus 2 reference channels) with a sampling frequency of 128 samples per second. Some advantages of using the Emotiv EPOC is its low cost, good signal-to-noise ratio, and ease of use (Duvinage et al., 2013). In addition, the EPOC has shown satisfactory results in diverse research studies in emotion recognition (Ramirez and Vamvakousis, 2012), brain computer interface (Holewa and Nawrocka, 2014), and cognitive workload (Lim et al., 2015). Figure 2 presents the headset and the channel positions based on the international 10–20 EEG system of electrode placement. Channel locations correspond to: AF3, F7, F3, FC5, T7, P7, O1, O2, P8, T8, FC6, F4, F8, AF4, M1, and M2. M1 is used as the ground reference channel for measuring the voltage of the other channels, while M2 is used as a feed-forward reference point to reduce external electrical interference (Badcock et al., 2015). A saline solution was employed to reduce the electrode impedance and facilitate sensitivity between each electrode and the scalp (Duvinage et al., 2013).

#### 2.6.1. EEG Pre-processing

EEG pre-processing was performed in Matlab (version 2018b, The MathWorks Inc.) by using custom software and the EEGLab toolbox (Delorme and Makeig, 2004). Baseline correction was performed by subtracting the corresponding mean from a pretrial (200ms) period from each channel. Then, EEG signals were band-pass filtered between 2 and 43 Hz using a FIR filter, which helps remove high-frequency artifacts and low-frequency drifts. Electrode movement artifacts were manually removed from the data; these artifacts produce large spikes that are several orders of magnitude bigger than the neural response produced by EEG. Artifacts from eye blinks and movements were corrected using the multiple artifact rejection algorithm (MARA) which evaluates ICA-derived components (Winkler et al., 2014).

#### 2.6.2. Feature Extraction

Feature extraction was carried out using spectral analysis. First, the power distribution from each channel was studied by transforming the EEG into power spectral density (PSD) using a fast-Fourier transform (FFT) and using 10-s windows with 50% overlapping windows multiplied by the Hamming function to reduce spectral leakage (Chaouachi et al., 2011). Second, from each window, the EEG channels were decomposed into sub-bands: delta (2–4 Hz), theta (4–8 Hz), alpha (8–12 Hz), beta (12–30 Hz), and gamma (30–40 Hz). Third, the PSD results of each frequency band were normalized (1/f) to obtain the relative PSD of each band to the baseline time period. This normalization helps to make quantitative comparisons of power across frequency bands (Cohen, 2014). Finally, the resulting PSD values in each band were averaged to obtain the power spectral features used for classification.

#### 2.6.3. Feature Selection

Feature selection was carried out to reduce the number of features and build a more accurate learning model. The selection criteria was based on the joint mutual information algorithm (JMI), this method ranks the features with the largest mutual information (MI) that produces most of the MI between the feature vector and the class label (Yang and Moody, 1999). The reason to choose JMI is that it presents better tradeoff in terms of accuracy, stability, and flexibility than other ranking methods (Brown et al., 2012; Rojas et al., 2019b). A disadvantage of this method is the fact that there is no stopping criteria to reach the best subset of features, and the user needs to select the number of features from the ranking list to form the optimal subset.

#### 2.6.4. Classification

The classification task is to determine the level of mental effort based on the recorded EEG signals from each participant. To identify the four levels of mental effort, we used the linear discriminant analysis (LDA) algorithm for offline analysis. The reason to choose LDA is because it is the most popular classifier in brain computer interface (BCI) research due to its good performance and low computational cost, attributes needed for the development of an on-line assessment of mental effort in our future work. To measure the classifier's performance, the data was divided into two parts with 70% for training and the remaining 30% used for testing and to report generalization performance. *k*-fold cross validation (*k* = 10) was performed on the training set; the training set was randomly divided into *k* partitions. Then, *k*-1 partitions are used to fit the learning model and the remaining partition used to validate the model, this process is repeated *k* times, and each time using a different partition to validate the model. The final generalization results are presented as the average and standard deviation on the 30% untouched test set.

### 2.7. Validation of Experimental Design

In order to validate the experimental conditions, the response to the ATWIT questionnaire and the heart rate information were analyzed. The research hypothesis of this study is that different levels in the quality of information (delay and dropout) significantly affect the perceived mental effort of the participants during the experimental task. In order to corroborate the research hypothesis and the design of this experiment, a repeated measures model was used to appraise statistical difference for the four different experimental conditions. Therefore, it was expected that the level of mental effort in each condition is significantly different and this difference can be observed by the subjective and objective metrics. A *p* value that is >0.05 was not considered statistically significant.

ATWIT scores and heart rate data were tested for normality using the Shapiro-Wilk test. Both tests showed that the data significantly deviated from a normal distribution, *p* = 0.01 for ATWIT scores and *p* = 0.033 for heart rate. Then, a logarithmic transformation was applied to reinforce the linearity of both data, which resulted in meeting the normality assumption (*p* > 0.05) after a subsequent normality test for both data. However, after checking normality visually using Q–Q plots (quantile–quantile plots), the distribution of both data was non-normal. Therefore, the non-parametric Friedman test was applied to both data for testing the difference between experimental conditions and Wilcoxon signed ranks test as *post-hoc* test.

**Figure 3:**
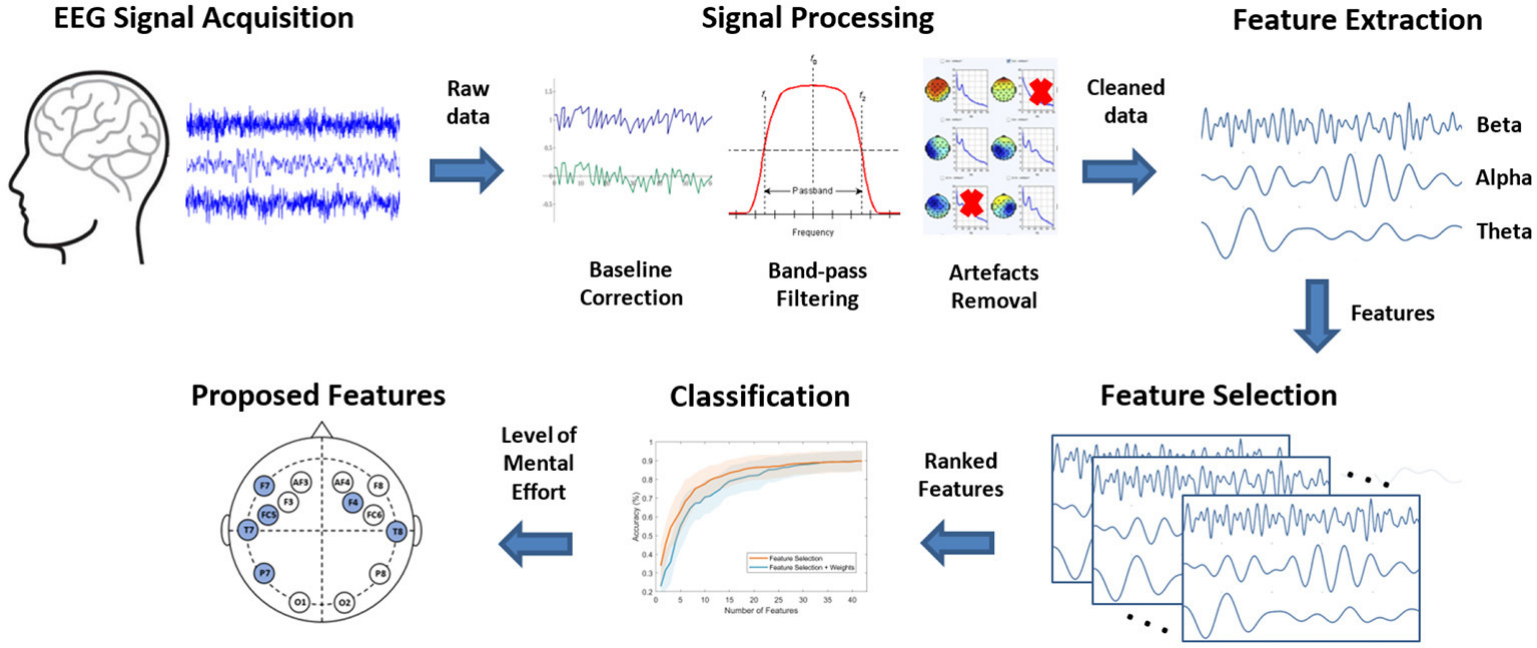
Analysis workflow used in the present study.

Figure 3 illustrates a summary of the analysis workflow used to obtain the results presented in this study. First, acquired EEG signals are cleaned through a series of signal processing techniques, then decomposition (feature extraction) of EEG signals into sub-bands (beta, alpha, theta) is carried out. The obtained features from each participant are then ranked using a feature selection technique. Each rank is then evaluated using an LDA classifier. Finally, the list of most prominent features contributing to the accuracy of the classifier are identified.

## 3. Results

### 3.1. Validation of Experimental Conditions

Two methods were used to evaluate the experimental design. First, the subjective workload assessment using ATWIT scores was evaluated for each experimental condition. Second, heart rate (HR) is used to corroborate the cognitive modulation with respect to each condition. The experimental assumption of this study is that in conditions with low quality communication (e.g., high delay and high dropout condition), the participants' perceived workload will be significantly different than in conditions with high quality communication (e.g., low delay and low dropout).

#### 3.1.1. ATWIT Scores

**Figure 4:**
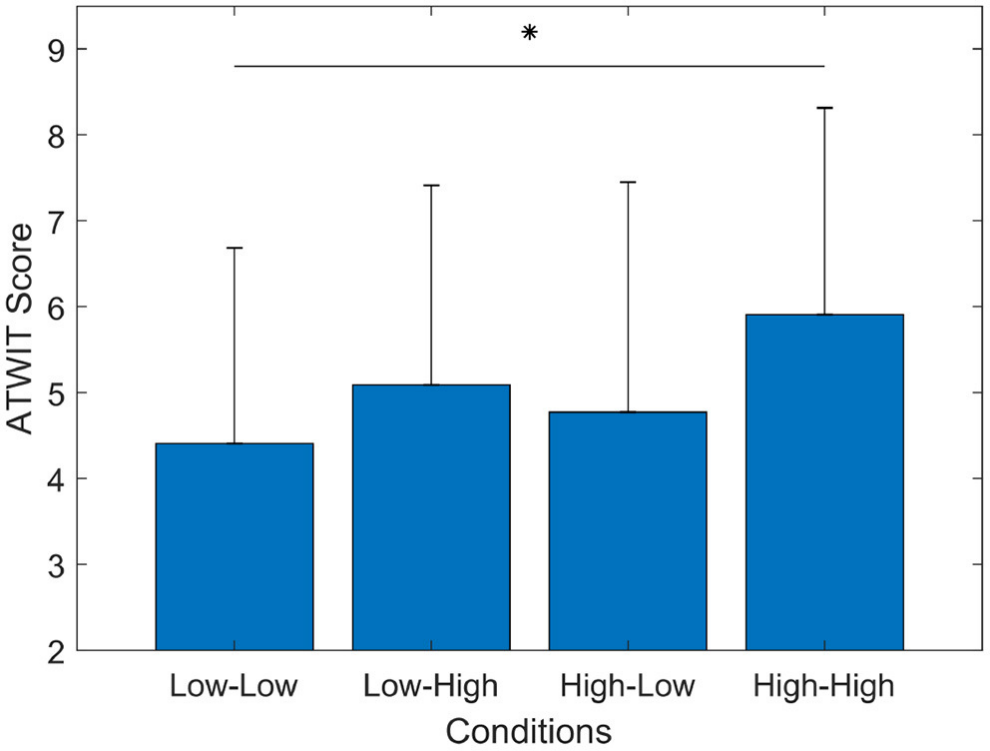
The bar graph represents the mean and standard deviation of ATWIT scores. The Wilcoxon test showed a significant increase of workload (*p* = 0.002). ^*^p < 0.0083.

Figure 4 shows the results of the subjective workload evaluation using ATWIT test. The recorded ATWIT response for each condition was averaged among the participants. The overall trend of subjects' perceived workload showed the lowest workload in the Low-Low condition (*mean* = 4.4, *std* = 2.2), medium workload in the Low-High (*mean* = 5.0, *std* = 2.3) and High-Low (*mean* = 4.7, *std* = 2.7) conditions, and the highest workload in the High-High (*mean* = 5.9, *std* = 2, 4) condition. Overall, ATWIT scores showed an increase of perceived workload in experimental conditions with low quality communication compared to experimental conditions with high quality of communication.

A Friedman test of differences among repeated measures was carried out to examine changes in ATWIT scores under the four conditions. This test was used with the following research hypothesis *Ho: There are no significant differences between the mean ATWIT scores among the experimental conditions*. In other words, the distribution of the answer to the ATWIT questionnaire is independent of the experimental condition (no difference in perceived workload). A statistically significant difference in perceived workload depending on the experimental conditions [χ^2^(*n* = 16) = 10.471, *p* = 0.015] was obtained. *Post-hoc* tests using multiple two-sided Wilcoxon signed-rank tests were performed with Bonferroni correction applied, resulting in a significance level set at *p* < 0.0083. There were no significant differences between the Low-Low and Low-High (*p* = 0.178), the Low-Low and High-Low (*p* = 0.502), the High-Low and Low-High (*p* = 0.303), the High-High and Low-High (*p* = 0.025), or the High-High and High-Low (*p* = 0.011) conditions. However, this statistical test showed a significant increase (*p* = 0.002) in perceived workload as declared in the ATWIT scores by the participants in the Low-Low and High-High scenarios.

#### 3.1.2. Heart Rate Information

**Figure 5:**
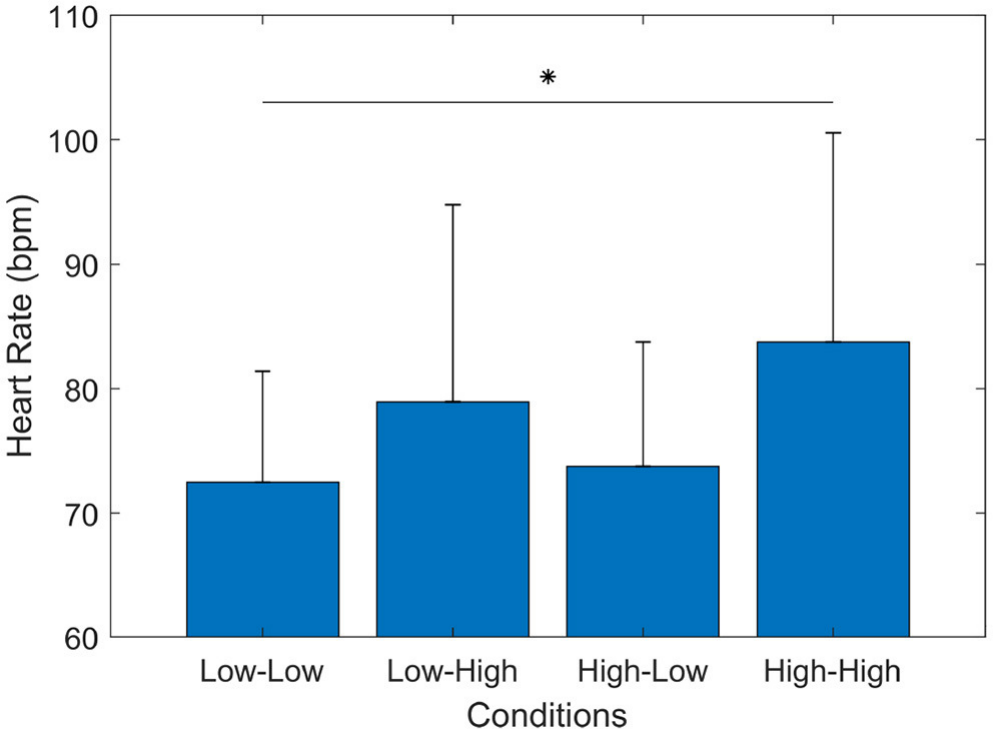
Subjects' heart rate (HR) in beats per minute (bpm). The bar graph represents the mean and the standard deviation of participants' HR during the four experimental conditions. The Wilcoxon test showed a significant increase between High-High and Low-Low (*p* = 0.003) conditions. ^*^p < 0.0083.

Another metric used to validate the experimental design was the participants' heart rate (HR). Figure 5 presents the results of the heart rate value between the four different conditions in the experiment. Heart rate has been shown to be a physiological indicator directly related to mental workload (Luque-Casado et al., 2016). In this case, the experimental assumption (refer to section methods) was that delay and dropout of information affect the cognitive workload of the participants and this can be observed by measuring the participants' heart rate. Overall, the results showed that during the Low-Low condition the participants exhibited the lowest HR (*mean* = 72.47, *std* = 8.9), medium HR during the Low-High (*mean* = 78.94, *std* = 15.83) and High-Low (*mean* = 73.76, *std* = 9.9), and the highest HR (*mean* = 83.73, *std* = 16.8) during the High-High condition.

A Friedman test was carried out to examine changes in heart rate under the four conditions. The Friedman test on the heart rate information revealed a significant difference among the scenarios [χ^2^(*n* = 16) = 15.60, *p* = 0.001]. *Post-hoc* tests using multiple two-sided Wilcoxon signed-rank test with Bonferroni correction applied showed that the participants' heart rate in High-High conditions increased statistically significant (*p* < 0.0083) compared to the heart rate in Low-Low (*p* = 0.003), while in the other conditions there were no significant differences between the Low-Low and Low-High (*p* = 0.039), the Low-Low and High-Low (*p* = 0.408), the High-Low and Low-High (*p* = 0.079), the High-High and Low-High (*p* = 0.023), or the High-High and High-Low (*p* = 0.01) conditions. These results showed that in experimental conditions with low quality communication (e.g., High-High) the participants' heart rate increased significantly, which also suggest an increase in cognitive workload as a result.

These two results (ATWIT and HR) validate the assumption that the different cognitive demands are affected due to the experimental conditions. In addition, high delay and high dropout exhibited the most significant increase in ATWIT score and heart rate, which suggests that this experimental condition induced the highest cognitive demand in the experiment. On the other hand, experimental conditions with low delay and low dropout exhibited the lowest ATWIT scores and lowest heart rate, which suggest that it produced the least cognitive demand in the experiment.

### 3.2. Evaluation of EEG Indicators

Based on the indicators identified in the literature, this study explores the classification performance using only three frequency bands: Theta (4–7.5 Hz), Alpha (8–12 Hz), and Beta (13–35 Hz).

#### 3.2.1. Reference Values

First, the indicators are investigated separately to obtain a reference performance value. Table 3 presents the grand average results from the classification task using each indicator. The results represent the classification accuracy (in percentage %) and standard deviation using each indicator separately. Each indicator was obtained from each channel. The highest overall accuracy (75.99 ± 6.48%) was achieved using the Beta band, while the lowest accuracy (49.10 ± 5.92) was obtained with Alpha band only.

**Table 3:** Reference values for classification accuracy and standard deviation (std) using LDA.

	**Power bands**			**Ratios**		
	**Theta**	**Alpha**	**Beta**	**Theta/Beta**	**Beta/(Alpha + Theta)**	**Theta/Alpha**
Accuracy	60.28	53.13	69.89	55.50	56.44	49.10
Std (±)	8.16	10.12	6.48	8.51	8.36	5.92

*Results are presented in percentage*.

#### 3.2.2. Feature Selection Evaluation

**Figure 6:**
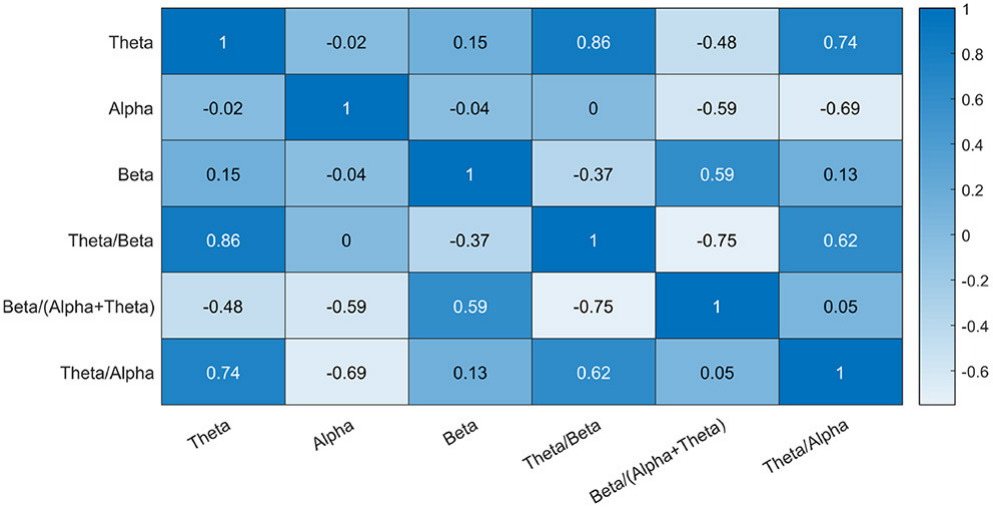
Strength and direction of correlations among the EEG features.

Second, using a feature selection method based on mutual information, to identify any kind of statistical dependency between variables, a subset of indicators was obtained. In this step, only the power bands (Theta, Alpha, Beta) were used in the feature selection process to avoid introducing correlated variables into the subset of indicators. Figure 6 presents the correlation analysis of all the indicators. In this figure, it is possible to observe that all the ratios (e.g., theta/beta) are highly correlated with the power bands (e.g., beta, alpha). Therefore, removing these variables from the analysis will make the feature selection more efficient.

The objective of feature selection is to find a good representation of the data, improve estimators' performance by reducing the dimensionality of the data and eliminating redundant and irrelevant data from each participant's data (Rojas et al., 2019b). After applying joint mutual information (JMI), the features were ranked according to the their relevance to the class label. This process returned 16 ranks (one rank per participant), each rank contains the ranking of 42 features (e.g., 3 indicators ^*^ 14 channels = 42 features). These ranks represent the importance of each indicator and channel with respect to the class label from each participant. However, a limitation of this process is that each rank is different from one another, which complicates direct comparisons between participants.

**Figure 7:**
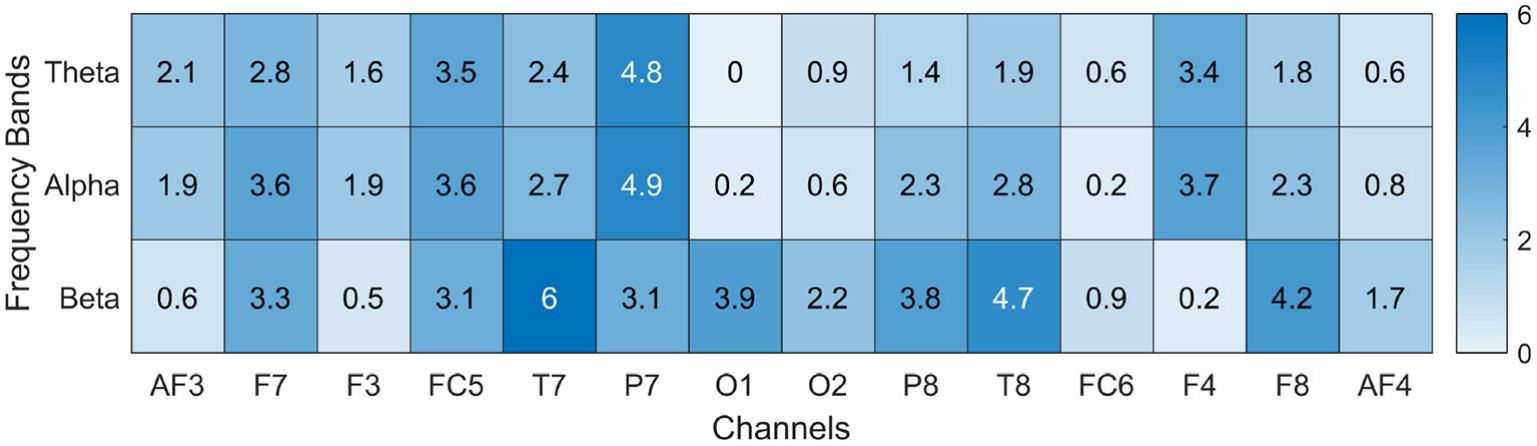
Frequency of appearance of each indicator. Each indicator was weighted according to its ranking (e.g., rank = 1 weight = 1, rank = 2 weight = 0.9) and number of occurrences in the list.

In order to obtain a subset of common features that potentially describe most of the data for all the participants, the frequency of appearances and ranking position were considered for each feature and for each participant. To achieve that, a new list containing the top 10 features from each rank were chosen (160 features in total). Then, the number of appearances of each feature in the list was counted and a weight [from 1 (most important) to 0.1 (least important)] for its position in each rank was given. For example, a feature (Theta in T8) appeared two times in the list (i.e., this feature was in the top 10 features only in two participants), in the first rank it appeared in the first position (*weight* = 1.0) and in the second rank in the seventh position (*weight* = 0.4); thus, its total value is 1.4 (please refer to Figure 7).

The complete results obtained during the weight process mentioned above is presented in Figure 7. Beta band in channel T7 (BetaT7) exhibited the highest value from this list, it suggests that this feature is the most important feature in our sample population. On the other hand, features from channels O1, FC6, and AF4 in the Theta and Alpha bands showed the lowest value from this list, which suggest that these features are less important among the 16 ranks. Based on this frequency of appearance, a common set of features can be obtained across the participants, which represents the most relevant features in the data set.

#### 3.2.3. Classification Results

**Figure 8:**
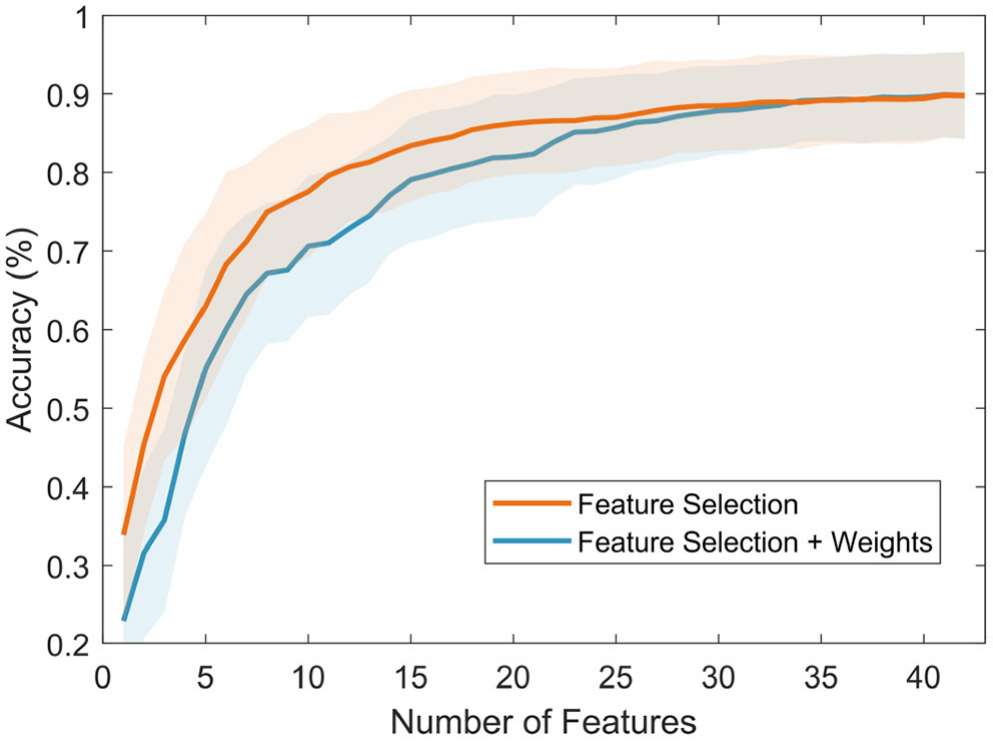
Classification results using the features ranked after the feature selection (using JMI) process (in orange) and after the feature selection + weights process (in blue).

Using both ranks, after feature selection (FS) and after application of weights (FS + weights), classification was carried out to obtain performance results and compare these with the obtained reference values (refer to section 3.2.1). Figure 8 presents the classification results of both methods. FS presented the highest accuracy (89.84 ± 5.60%) using the top 40 features (in total, 42 features), while the FS + weights method presented slightly lower accuracy (89.43 ± 5.47%) using the same number of features. It is to note, that using the top 10 features from both methods produced a higher accuracy (77.57 ± 8.39% for FS, and 70.60 ± 8.98% for FS + weights) than the highest reference accuracy value (69.89 ± 6.48) using any of the frequency bands and ratios separately, which was achieved using the 14 channels in the Beta band. Overall, both methods showed comparable results, which suggests that the group of common features across our sample population can be used as indicators of cognitive load in our experiment.

However, in order to identify the appropriate number of features to be used as indicators of workload in our experiment, a stopping criterion was introduced. This criterion is based on comparing consecutive classification results using two-sample *t*-tests. Using this method, the searching process is stopped when three consecutive non-statistically significant results were obtained. The final number of features is the one that produced the first non-statistically significant result. After this step, the appropriate number of features is 18 for FS (85.44 ± 6.70%) and 19 for FS + weights (82.83 ± 8.01%). These top 19 features are presented in Table 4.

**Table 4:** Top 19 features after feature selection and weight procedure (FS + weights).

**Ranking**	**Channel**	**Band**	**Ranking**	**Channel**	**Band**
1	T7	Beta	11	FC5	Theta
2	P7	Alpha	12	F4	Theta
3	P7	Theta	13	F7	Beta
4	T8	Beta	14	FC5	Beta
5	F8	Beta	15	P7	Beta
6	O1	Beta	16	F7	Theta
7	P8	Beta	17	T8	Alpha
8	F4	Alpha	18	T7	Alpha
9	FC5	Alpha	19	T7	Theta
10	F7	Alpha			

#### 3.2.4. Activated Cortical Areas

**Figure 9:**
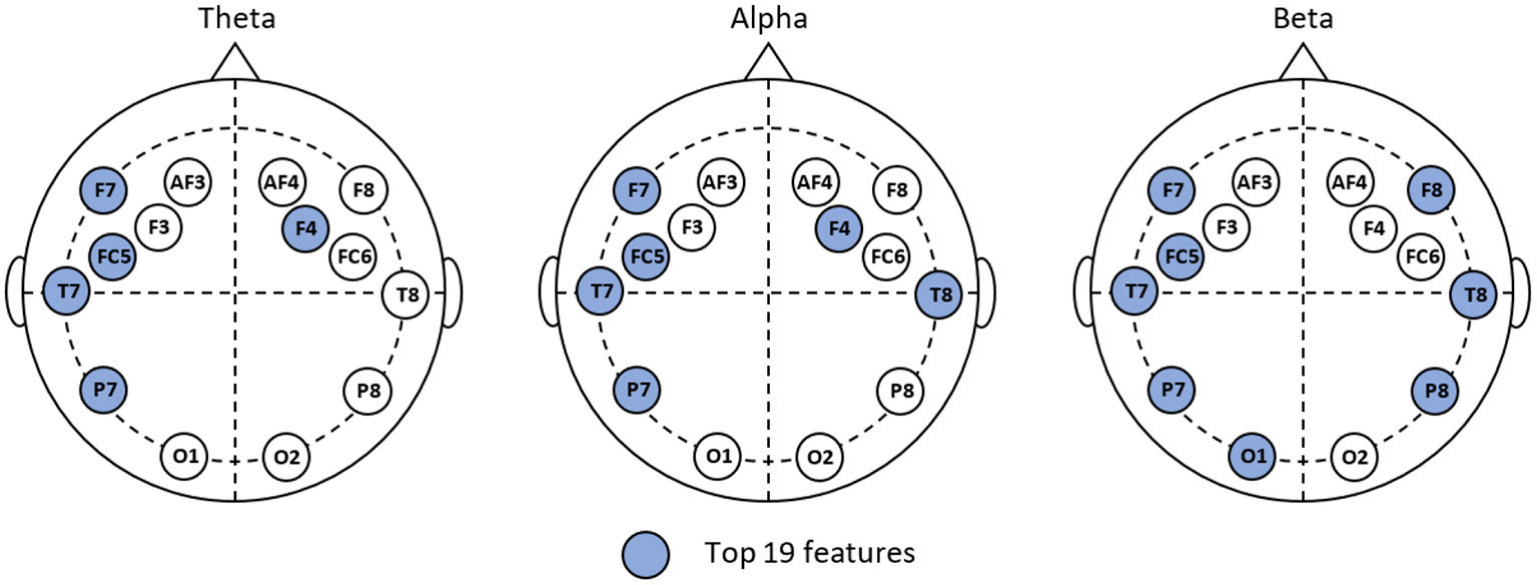
Cortical locations of the top 19 features in the three bands explored in this study.

The majority of features from the identified subset (top 19 features) are from the Beta band and the frontal area. Figure 9 presents the cortical location of each feature with respect to their frequency band (Theta, Alpha, Beta). Three channels (F4, F7, FC5) from the frontal area, one channel from the temporal area (T7) and one channel (P7) from the parietal area were obtained in the Theta band. In the Alpha band seven channels were identified, the same three channels in the frontal area (F4, F7, FC5), bilateral activation in the temporal area (T7, T8), and one channel (P7) from the parietal area. The Beta band exhibited the largest number of channel within the top 19 features in four cortical locations, in the frontal (F7, FC5, F8), bilateral activation in both the temporal (P7, P8) and parietal (P7, P8) cortex, and in the occipital area (O1). Another interesting finding is that most of the features in the top 19 corresponded to the left hemisphere.

#### 3.2.5. Evaluation of the Weight Process

Two more filter feature selection methods were used to evaluate the weight process to capture the most common features across the sample population. These two techniques are Information Gain (InfoGain) and student's *t*-test, their criteria to rank each feature are entropy and statistical based (Novaković, 2016), respectively. These two feature selection techniques were implemented and a group of 16 different ranks (i.e., one rank per subject) was obtained from each technique. Then, the weight process was applied to each technique separately using the top 10 features from each participant. Please refer to section 3.2.2 for a more detailed description.

**Figure 10:**
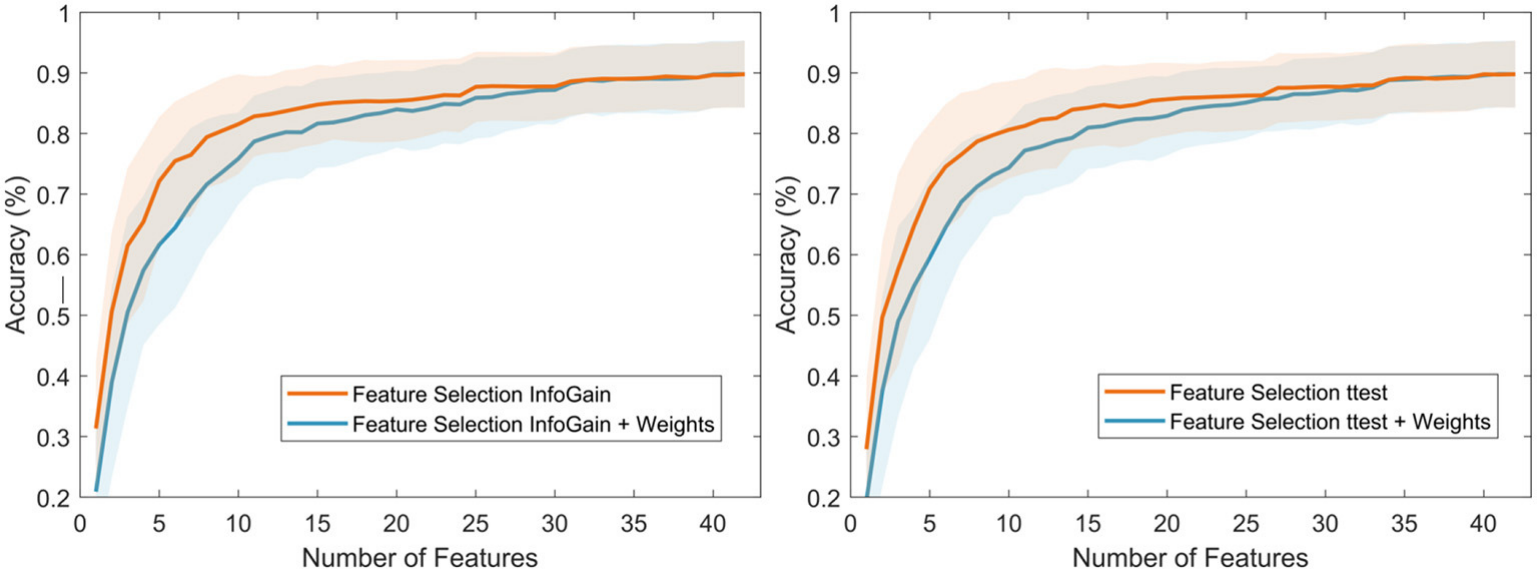
Classification results using Information Gain **(left panel)** and *t*-test **(right panel)**.

Figure 10 presents the classification results of both techniques using LDA. It was expected that each technique will produce different rankings and different classification results. This is mainly due to the different ranking strategies followed by different techniques. In addition, similar to the JMI technique, InfoGain and *t*-test lack a stopping criterion to obtain the best feature subset; therefore, three consecutive non-statistically significant results were used to stop the searching process for each method. For the InfoGain method, the stopping criterion led to 16 features (85.06 ± 6.04%) and 19 for InfoGain + weights (83.36 ± 6.63%). For the *t*-test method the stopping criterion led to 17 features (84.40 ± 6.74%) and 27 for *t*-test + weights (85.77 ± 5.38%).

These results highlight that the weight process captures the most common features among the sample population. Introducing the weight process after feature selection allows the classifier to maintain a comparable performance than the reliance on individual rankings for each participant. Therefore, the use of common features not only facilitates making comparisons across subjects but also reduces the complexity of the analysis by focusing on a smaller set of features.

### 3.3. Sensitivity of EEG Indicators

**Figure 11:**
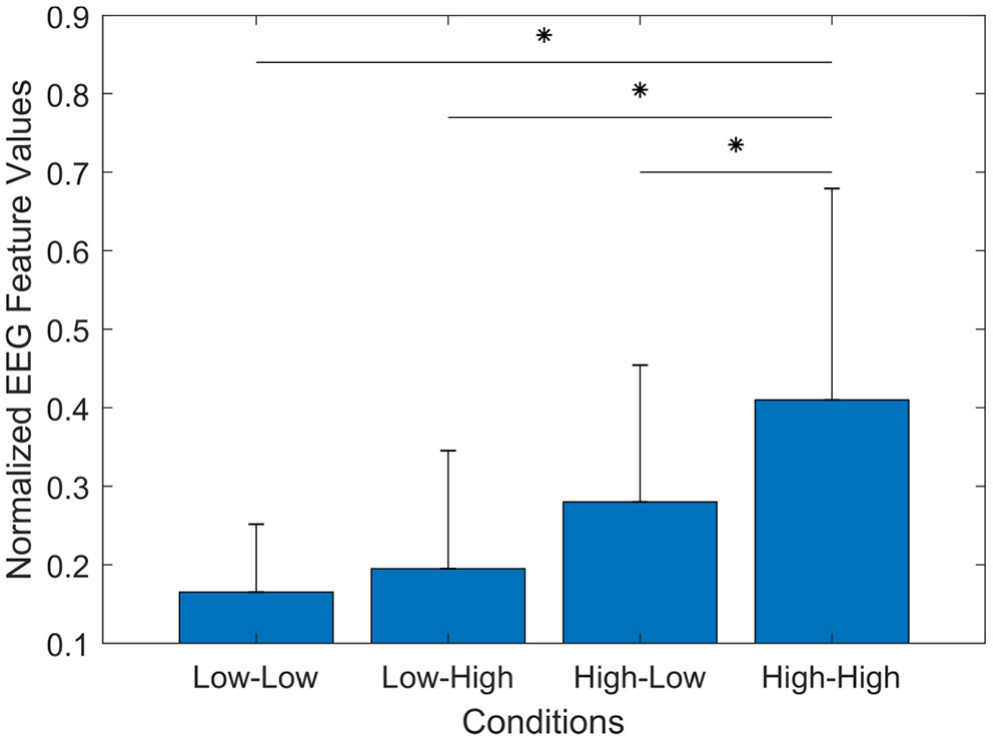
The bar graph represents the mean and standard deviation of the proposed set of EEG features. The Wilcoxon test showed a significant increase of workload the Low-Low and High-High (*p* = 0.000), the High-High and Low-High (*p* = 0.005), and the High-High and High-Low (*p* = 0.000) conditions. ^*^p < 0.0083.

In order to examine the sensitivity of the proposed set of EEG indicators to differentiate between the four experimental conditions (i.e., four levels of workload), a test for differences was conducted using the Friedman Test. This test was used with the following research hypothesis *Ho: There are no significant differences between the mean EEG values among the experimental conditions*. In other words, the distribution of EEG values is independent of the experimental conditions (the EEG indicators do not capture a difference in workload). Figure 11 presents the results of this test.

The results exhibited a statistically significant difference in EEG values depending on the experimental conditions [χ^2^(*n* = 16) = 31.27, *p* = 0.000]. *Post-hoc* tests using multiple two-sided Wilcoxon signed-rank tests were performed with Bonferroni correction applied (*p* < 0.0083). There were no significant differences between the Low-Low and Low-High (*p* = 0.030), the Low-Low and High-Low (*p* = 0.01), or the High-Low and Low-High (*p* = 0.026) conditions. However, this statistical test showed a significant difference in the Low-Low and High-High (*p* = 0.000), the High-High and Low-High (*p* = 0.005), and the High-High and High-Low (*p* = 0.000) conditions. This result suggests that using the proposed set of EEG features presents higher sensitivity to measure cognitive load during our experiment, than the ATWIT questionnaire and the heart rate.

## 4. Discussions

The primary goal of the current investigation was to examine different EEG indicators for the objective assessment of cognitive workload. An experiment was designed to modulate the participants' perceived workload. EEG indicators of spectral powers at different cortical locations (based on theta, alpha, and beta bands) were compared and investigated. Using a feature selection technique, the most important features were obtained for each subject, then a weight procedure was applied to identify a set of common features across our sample population. The identified set of features represents a group of possible EEG indicators for the objective assessment of cognitive workload.

The experimental conditions and overall assumption of the experiment were validated. The research hypothesis about the use of delay and dropout to modulate the participant's perceived workload was confirmed by using statistical analysis performed on both the averaged ATWIT response and heart rate (HR) data. The overall trend exhibited that the participants faced a significantly higher (*p* < 0.0083) cognitive workload during the high delay and high dropout (High-High), a similar trend was also observed using the EEG indicators. This finding suggests that the increase of participants' cognitive workload in scenarios with high delay and high dropout reflects the difficulty in understanding and identifying new information after the loss of an already-familiar scenario. This is in line with previous studies on the relationship between information quality and workload. For instance, in an experiment to study the effect of audio communication latency on cognitive workload (Krausman, 2013), it was found that increased audio communication latency led to increased cognitive workload and lower task accuracy. Similarly, increased workload has been reported in participants after the use of automation in teleoperated systems, where participants face new information after the use of automation to complete a task (Chen et al., 2017).

The weighting process after feature selection (FS + weight) helped obtain a common set of features across our sample population. In the machine learning and the data mining literature, feature selection is an important preprocessing step in regression and classification problems (Vergara and Estévez, 2014). An advantage of using feature selection in comparison with other dimensionality reduction methods (e.g., PCA) is that feature selection does not alter or transform the data; thus, attempting to understand the underlying process that produced a given classification result can be achieved (Bennasar et al., 2015). In our experiment, although feature selection was used to determine the most important features for each subject and also to identify the irrelevant features to be discarded, it produced sixteen different rankings that made it difficult to deduce a common set of features. Thus, the weighting process helped determine a common set of features by using the individual rankings of each feature from each participant. The resulting set (Table 4) represents the most frequent features in the complete feature set. It is worth mentioning that by using the ranked features according to their relevance to the class label, the weight process retains useful intrinsic groups of interdependent features, which helped avoid redundant and irrelevant features in the FS process. This common set of features (top 19) represents less than half (~45%) of the total number of features.

The common set of features showed objective confirmation of the different levels of perceived workload during the classification task. The classification task exhibited a much better performance (82.23%) using the top-19 features than any of the reference values (Table 3) using each indicator separately. In addition, the obtained feature set represents a combination of well-known EEG power bands that have been linked to cognitive workload as identified in our literature review. These frequency bands (theta, alpha, beta) are generally associated with a different dimension of workload (e.g., attention, vigilance, or mental fatigue). For instance, theta band has been successfully used to study mental fatigue and alertness (Gevins et al., 1995; Kamzanova et al., 2014), alpha band has been employed to assess mental vigilance, attention and alertness (Antonenko et al., 2010; Borghini et al., 2012; MacLean et al., 2012), while beta band has been used to study visual attention or short-term memory (Tallon-Baudry et al., 1999; Wróbel, 2000; Palva et al., 2011). Therefore, using a combination multiple frequency bands will make the assessment of workload more robust to other intrinsic cognitive processes that are carried out simultaneously. This is particular important, since our experiment reflects a multitasking environment where different dimensions are present at the same time, e.g., navigation while maintaining orientation, or planning while maintaining communication distance with the alpha vehicle.

The identified feature set also helped identify the most relevant cortical areas associated with the assessment of cognitive workload in our experimental task. The majority of identified channels are from the frontal, temporal, and parietal regions, cortical areas that have been associated to cognitive workload in previous studies. For instance, increase in theta band power over the frontal cortex has been associated with an increase in task difficulty and use of more working memory resources (Parasuraman and Caggiano, 2002). Suppression of Alpha power has been observed in the parietal and occipital areas during increase of mental workload (Mazher et al., 2017; Puma et al., 2018). Increase in Beta power over the parietal and occipital cortical regions has been observed during visual working memory tasks (Mapelli and Özkurt, 2019). In addition, bilateral activation was identified in the beta band. These activations were found in the frontal (F7 and F8), temporal (T7 and T8), and parietal (P7 and P8) areas. While in the alpha band, a bilateral activation was only found in temporal areas (T7 and T8). The observed bilateral activation in different cortical areas suggests that there is is no single brain region or hemisphere that solely responds to mental workload. In addition, as many other cognitive tasks, the brain functions as a system rather than separated brain areas working independently (Rojas et al., 2016).

We acknowledge that this study presents some limitations that should be addressed in our future research. The use of a small number of electrodes to monitor the cortical activity restricts our ability to make generalizations to other cerebral regions from the proposed set of EEG features. Advantages of using the Emotive EPOC is that it is less uncomfortable to be worn for longer periods of time and less unpleasant for participants since it uses dry electrodes. However, in future research a larger number of electrodes to record activity in more areas of the cerebral cortex should be considered. Another limitation of this study is that each sensor modality was analyzed separately to study workload. Debie et al. (2019) highlighted the disadvantages of using a single sensor modality to capture changes in cognitive workload. For instance, a given measure may respond to a particular task (e.g., attention or engagement) but may fail to capture workload change in other tasks (e.g., working memory or mental fatigue). Thus, combining multiple sensors can measure different aspects of workload and can potentially complement one another to provide a better assessment of cognitive workload in multitasking situations.

Finally, the results of this study expand earlier findings from previous research of cognitive workload assessment using EEG. However, direct comparisons with other studies are difficult because of the use of different experimental conditions, EEG acquisition system, sampled population and with different demographics, validation methods, and classification models (Rojas et al., 2017a). Therefore, the contributions of this study can be summarized as follows: (1) it offers an exploratory study that aims to compare different EEG indicators identified in the literature for the objective assessment of cognitive workload; (2) it introduces a framework to extend the feature selection process to identify the most important features among the sample population; and (3) it presents a group of features (EEG power bands and cortical regions) as possible indicators for the objective assessment of cognitive workload in multitasking environments.

## 5. Conclusions

This study investigated different EEG power bands to identify a set of indicators that can be used for the objective assessment of cognitive workload. Results showed that our experimental study was valid at increasing mental workload in the participants as measured by three metrics (ATWIT, HR, and EEG). The use of a weighting process after feature selection (FS + weights) helped identify common features across all participants. In addition, a set of indicators (including EEG power bands and cortical regions) was identified as objective metric of workload in our multitasking environment. The proposed set of indicators exhibited higher sensitivity to various levels of cognitive workload than the subjective metric (ATWIT) and the physiological measure (heart rate). Finally, future research will adopt the proposed EEG indicators to trigger adaptive automation to maintain performance in human-swarm teaming.

## Data Availability Statement

The datasets for this article are not publicly available due to confidentiality requirements with our funder. Requests to access the datasets should be directed to Hussein Abbass: h.abbass@unsw.edu.au.

## Ethics Statement

The studies involving human participants were reviewed and approved by University of New South Wales (UNSW) Research Ethics Committee. The patients/participants provided their written informed consent to participate in this study.

## Author Contributions

RF and ED carried out the experiment. ED implemented the simulation environment. RF analyzed the data and wrote the manuscript in consultation with HA. HA conceived the study and was in charge of overall direction and planning. JF, MB, KK, SA, and MG offered domain knowledge in the design of the system, discussed the results, and commented on the manuscript.

### Conflict of Interest

The authors declare that the research was conducted in the absence of any commercial or financial relationships that could be construed as a potential conflict of interest.
